# Morphological Responses of *Viola* Accessions to Nutrient Solution Application and Electrical Conductivity

**DOI:** 10.3390/plants11111433

**Published:** 2022-05-27

**Authors:** Endre Kentelky, Zsolt Szekely-Varga, Irina M. Morar, Mihaiela Cornea-Cipcigan

**Affiliations:** 1 Department of Horticulture, Faculty of Technical and Human Sciences, Sapientia Hungarian University of Transylvania, Calea Sighișoarei 2, 540485 Târgu Mureș, Romania; kentelky@ms.sapientia.ro; 2 Department of Forestry, Faculty of Horticulture, University of Agricultural Sciences and Veterinary Medicine of Cluj-Napoca, 400372 Cluj-Napoca, Romania; 3 Department of Horticulture and Landscaping, Faculty of Horticulture, University of Agricultural Sciences and Veterinary Medicine Cluj-Napoca, 400372 Cluj-Napoca, Romania; mihaiela.cornea@usamvcluj.ro; 4 Laboratory of Cell Analysis and Spectrometry, Advanced Horticultural Research Institute of Transylvania, University of Agricultural Sciences and Veterinary Medicine of Cluj-Napoca, 400372 Cluj-Napoca, Romania

**Keywords:** growth, multivariate statistics, nutrient solution, pansy

## Abstract

Growing of ornamental flowering plants represents an important sector of horticulture. *Viola* × *wittrockiana* (garden pansy) is used in garden beds and borders due to their colorful blooming, which occurs in early spring and late autumn. Nowadays, breeders focus on applying different nutrient solutions to improve the quality, flowering, and ornamental properties of plants, yet electrical conductivity (EC) level is an important fact to know. It is known that higher EC levels can inhibit plants’ growth. In the present study, pansy seedlings were subjected to different EC nutrient solutions 1 (control), 2, 3.5, 5, and 6.5 mS cm^−1^ EC to assess the positive or negative effects regarding the plant’s growth and development. The results indicated that an appropriate EC level of nutrient solution can have a positive effect on growth parameters, as well as on the flowering of plants. According to the hierarchical clustering, the used EC nutrient solutions significantly influenced the growth, number of shoots and leaves and the inflorescences number. From the present study results, it can be concluded that even though all EC levels increased growth parameters compared with control, the greatest results were obtained in plants under the effect of the 5 mS cm^−1^ of EC.

## 1. Introduction

Plant cultivation has always been focused on food production; however, breeders did not neglect the production of ornamental plants [1]. Floriculture is by far one of the most important sections of the horticulture sector, and it contains a wide range of products [2,3], such as cut flowers and greens, indoor and outdoor plants, bedding and garden plants [4,5]. Ornamental plants have a much-requested mark, and with an increasing demand, rapid innovations are needed [6] to obtain high quality products. For this reason, the floriculture sector is one of the most highly innovative branches of plant cultivation, with these innovations often being used in other areas of horticulture [1].

The genus *Viola* L. belongs to the family Violaceae, and it is native to Europe and western Asia, with 265 species [7].

*Viola* × *wittrockiana* Gams., commercially known as pansy is one of the most important biannual plants for garden beds and borders, also used as a culinary art [8,9]. Pansy flowers are colorful, and blooming occurs in early spring and late autumn, enriching the color of areas where planted [10,11]. Pansies have a complex hybrid origin, involving three species from the genus Violaceae, namely *Viola tricolor* L., *V. altaica* Gawl. and *V. lutea* Huds. [11,12].

Most of the commercial producers use different nutrient solutions to improve the quality of inflorescences, to obtain abundant and fully flowering plants. Therefore, nutrient solution influences the growth and productivity of plants [13]. By applying a higher quantity of these solutions, most of it flows into the soil, which may lead to soil degradation, namely acidification, salinization, nutrient imbalance, and irregular accumulation of nitrogen, phosphorus, and potassium [14,15], resulting in environmental pollution [16]. Furthermore, inadequate application of nutrients can have a negative influence on the soil pH level [17,18]. Growing media provides plants with nutrients, air, water, and physical support [19]. Also, growing media includes pH and electrical conductivity (EC) and due to the fact that these factors can have values that are too low or high, the growth and quality of plants can decrease [20]. Additionally, pH and EC levels of the growth media can be influenced by applying different nutrient solutions [11].

EC level is an important factor in nutrient solution and reflects the total content of macro—and microelements available for the plants [21]. High EC levels can inhibit the nutrient uptake by increasing osmotic pressure and salt stress, whereas low EC levels may lead to nutrient deficiencies and inhibition of growth and development of plants [22]. Salt stress can cause premature aging of the leaves and chloroplast and chlorophyll content reduction [23], which eventually leads to plant death [24,25]. High salinity stress makes it difficult for the plants to uptake nutrients and also water, due to a lower water potential in the soil, which leads to water shortage in plants [26,27]. The main ions responsible for salinity stress are Na^+^, K^+^, and Ca^2+^. Interaction of previously mentioned ions provides homeostasis to cells [28]. Potassium (K) is one of the most important plant nutrients, and is required to activate over 80 different enzymes responsible for plant processes, such as nitrate reduction, photosynthesis, and sugar degradation [29,30,31,32,33,34]. Furthermore, it was reported that K^+^ can decrease different stress effects, such as high light intensity and drought [35].

The present study investigated the effect of different EC levels 1 (control), 2, 3.5, 5, and 6.5 mS cm^−1^ EC on *Viola* × *wittrockiana* ‘Delta’ with three distinct colors, to improve the quality of plants by optimizing the nutrient solution level. Nutrient solutions are vital nutrients which are required by the plants for normal growth, development, and increased productivity. We aimed to determine which EC level is the most suitable for pansies growing, to reduce the unnecessary use of nutrient solutions and have a lower negative impact on the environment.

## 2. Results

### 2.1. Growth Media pH and EC Levels

**Figure 1:**
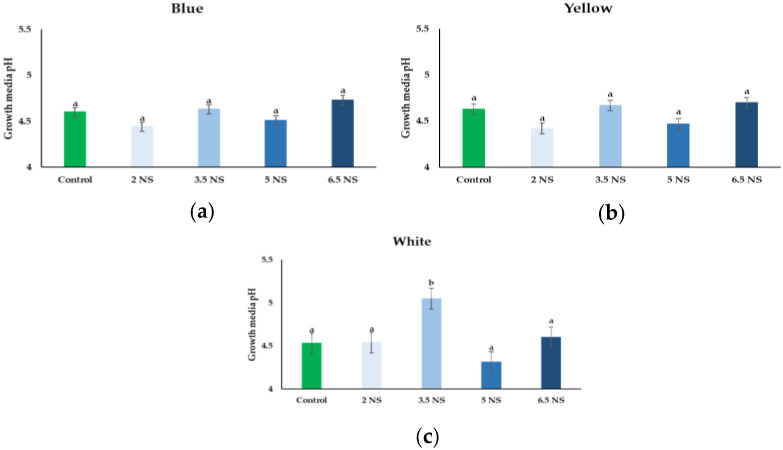
Effect of nutrient solution electrical conductivity levels 1 (control), 2, 3.5, 5, and 6.5 mS cm^−1^ EC on growth media pH of the selected *Viola* × *wittrockiana* seedlings: (**a**)—Blue, (**b**)—Yellow, and (**c**)—White. Bars represent the means ± SE (*n* = 40). Different letters indicate significant differences between treatments (*p* < 0.05).

No significant differences were noticed regarding the seedlings used (data not shown). The growth media pH did not show significant differences when treatments were applied (Figure 1). *Viola* × *wittrockiana* Blue and Yellow seedlings registered similar pH levels compared with control. The pH level at Blue seedlings was approximatively~4.58 (Figure 1a) and Yellow~4.6 (Figure 1b). However, an increased level was noticed under the effect of the 3.5 mS cm^−1^ EC treatment, where the average pH level was 4.53 at untreated plants and 5.05 at the 3.5 mS cm^−1^ EC (Figure 1c).

**Figure 2:**
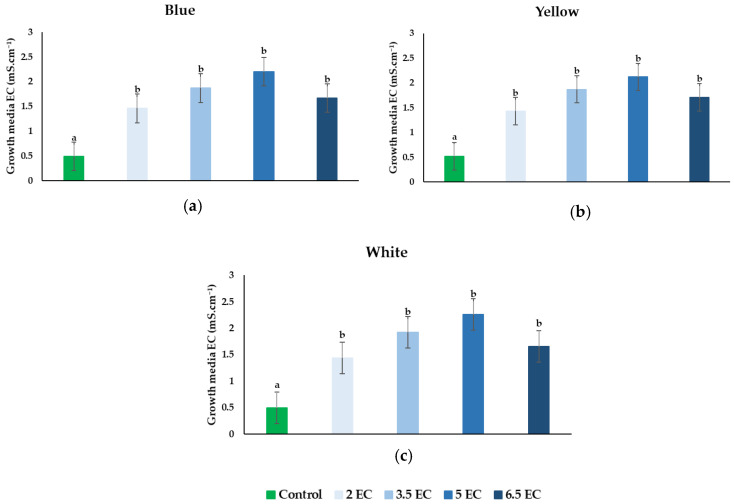
Effect of nutrient solution electrical conductivity levels 1 (control), 2, 3.5, 5, and 6.5 mS cm^−1^ ECon growth media EC of the selected *Viola* × *wittrockiana* seedlings: (**a**)—Blue, (**b**)—Yellow, and (**c**)—White. Bars represent the means ± SE (*n* = 40). Different letters indicate significant differences between treatments (*p* < 0.05).

Considering the EC of the growth media, it can be stated that the treatments influenced its levels (Figure 2). Analyzing Blue pansies, it can be observed that as the treatment level increased, the EC increased with it (Figure 2a). All four-treatment levels recorded significant differences compared with control. However, the EC levels increased with the treatment level, and a small decrease was observed at the 6.5 mS cm^−1^ EC treatment, insignificantly compared to the other treatments. Regarding the Yellow and White *Viola* × *wittrockiana*’s, similar results were obtained. Here again, significant increases were determined when comparing the treatments with control (Figure 2b,c). It is important to mention that however we irrigated and sprayed the plants with the above-mentioned nutrient treatments levels, the growth media EC level was lower than the applied solution.

### 2.2. Seedlings Growth

As expected, the growth of pansies was affected by the treatments in a positive way (Table 1). All four treatments (2, 3.5, 5, and 6.5 mS cm^−1^ EC) significantly increased the growth of the ‘Delta’ Blue seedlings, in some case with even 1.3 cm, compared with control. Significant increases were also determined at Yellow pansy plants subjected to treatments. Under the 5 mS cm^−1^ EC, treatment a greater increase compared with the other treatments was recorded. White *Viola* seedlings showed higher development compared with control under all treatments.

### 2.3. Shoot Number Affected by Treatments

Regarding the shoot number, the treatments greatly influenced the shoot appearance in a positive way, which led to a more compact plant (Table 1). Considering Blue pansies, it can be stated that all four treatments increased the shoot number compared with control; however, 5 mS cm^−1^ EC reported higher increases, almost two times, compared with the other treatments. Significant increases were also observed in Yellow pansies. Similar to the previous pansies, all treatments affected in a positive way the shoot number of the seedlings, even 5 mS cm^−1^ EC treatments reported significant increases compared with the other treatments (2, 3, and 6.5 mS cm^−1^ EC). Here again, the results showed that higher levels of electrical conductivity positively increased the shoot number, and in this case also seedlings from 5 mS cm^−1^ EC treatment determined higher increases compared with the other treatments. Seedlings under the 5 mS cm^−1^ EC treatment reported ~3.5 times more shoots than control and ~2 times more than the other three treatments.

### 2.4. Leaves and Inflorescences Number under the Influence of Treatments

As expected, the electrical conductivity level influenced the leaf appearance of seedlings (Table 1). From the results of Blue pansies, it can be stated that the treatments affected the number of leaves in a positive way. Two, 3.5, and 6.5 mS cm^−1^ EC treatments recorded approximately ~8 more leaves compared with control, and 5 mS cm^−1^ EC with ~11 leaves. However, all treatments reported higher increases compared with control, and seedlings under the 5 mS cm^−1^ EC treatment reported significant increases compared with the other three treatments. At ‘Delta’ Yellow, leaf number was also affected positively by the treatments. The seedlings subjected to EC treatments significantly exceeded the control, with 5 mS cm^−1^ EC having the best results in terms of leaf numbers compared with the other treatments. Regarding ‘Delta’ White seedlings, it can be noticed that all four treatments significantly increased the number of leaves, with no significance observed between the treatments.

**Figure 3:**
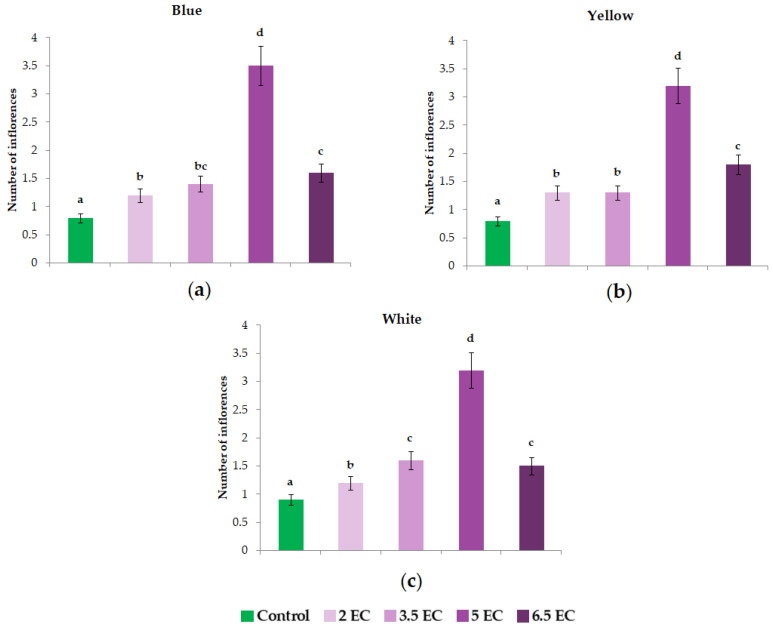
Effect of nutrient solution electrical conductivity levels 1 (control), 2, 3.5, 5, and 6.5 mS cm^−1^ EC on the inflorescences number of the selected *Viola* × *wittrockiana* seedlings: (**a**)—Blue, (**b**)—Yellow, and (**c**)—White. Bars represent the means ± SE (*n* = 40). Different letters indicate significant differences between treatments (*p* < 0.05).

The inflorescences number was affected by EC in a dose-dependent manner (Figure 3). In ‘Delta’ Blue seedlings, all four treatments positively influenced the inflorescences number; however, 5 mS cm^−1^ EC recorded a higher increase compared with the others, with approximately ~2 times more. In Yellow pansies, significant increases were observed at 5 and 6.5 mS cm^−1^ EC compared with control, although in this case, the 5 mS cm^−1^ EC treatment reported the greatest increase. Considering White pansies, significant differences were determined only at 5 mS cm^−1^ EC. Regarding the other treatments, no significant differences were noticed, except 3.5 and 6.5 mS cm^−1^ EC treatments, which presented similar results.

**Figure 4:**
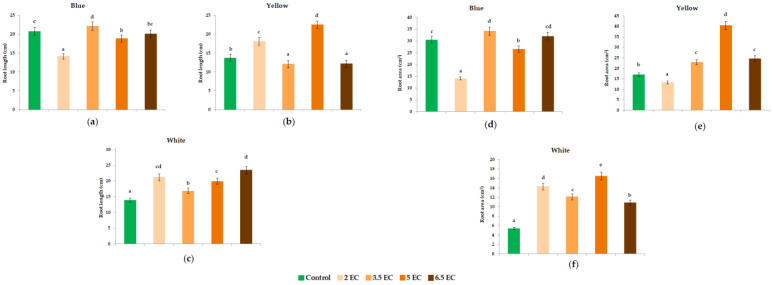
Effect of nutrient solution electrical conductivity levels 1 (control), 2, 3.5, 5, and 6.5 mS cm^−1^ EC on root length (cm) (**a**–**c**) and root area (cm^2^) (**d**–**f**) of selected *Viola* × *wittrockiana* seedlings. Bars represent the means ± SE (*n* = 40). Different letters indicate significant differences between treatments (*p* < 0.05).

The root length and area have been significantly affected by EC treatments in a dose-dependent manner (Figure 4). In the Blue seedlings, significant differences were noticed in the 2 mS cm^−1^ EC treatment with the lowest root length and area, whereas the highest length and area were shown in the 3.5 and 6.5 mS cm^−1^ EC treatments compared with control. In Yellow pansies, an increase in root length was noticed in 5 mS cm^−1^ EC treatments, whereas the lowest were determined with 3.5 and 6.5 mS cm^−1^ EC treatments, respectively. Conversely, significant differences in root area were noticed under the 2 mS cm^−1^ EC treatments with the lowest root architecture trait, compared with 5 mS cm^−1^ EC treatments which positively influenced the root development. Similarly to Blue seedlings, a positive influence was noticed in White pansies under the 6.5 mS cm^−1^ EC treatments, whereas the lowest root length was observed with the 3.5 mS cm^−1^ EC treatments. Regarding the root development, significant differences were noticed under the 5 mS cm^−1^ EC treatments, whereas the lowest were observed in 6.5 mS cm^−1^ EC treatments.

The plant volume was significantly affected by EC treatments in all Viola varieties (Table 1). In the White cultivars, significant differences were observed in the 3.5 mS cm^−1^ EC with the lowest plant volume, whereas the highest volume was noticed with the 5 and 6.5 mS cm^−1^ EC treatments compared with control. In Blue pansies the highest increase in plant volume was noticed with 5 mS cm^−1^ EC treatments and the lowest with 2 mS cm^−1^ EC compared with control. Regarding the plant volume in Yellow pansies, the lowest values were observed with 2 and 3.5 mS cm^−1^ EC with no significant differences between treatments, whereas the highest was observed with 5 mS cm^−1^ EC treatments.

### 2.5. Relationship between the Vegetative Parts and Number of Inflorescences Appearance under the Influence of Different EC Nutrient Solutions

**Figure 5:**
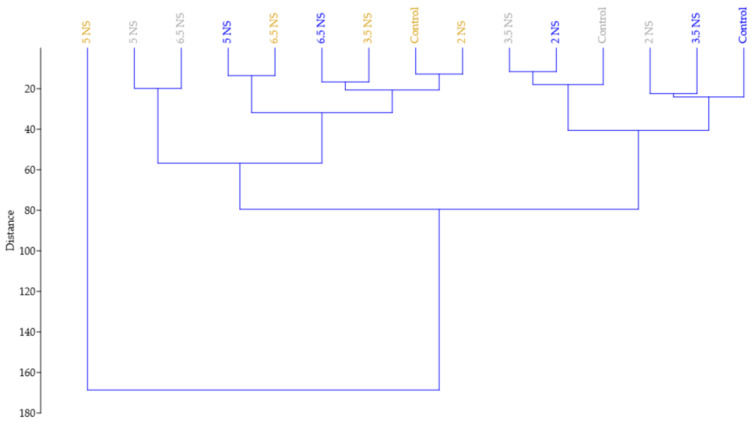
Hierarchical clustering of the *Viola* × *wittrockiana* seedlings based on the growth and number of inflorescences. Colored designation represents the type of seedlings (grey—White *Viola* seedlings; blue—Blue *Viola* seedlings; golden—Yellow *Viola* seedlings).

A cluster analysis was performed to differentiate the plants’ growth and applied treatments. For the analysis, the dataset incorporating the growth of plants along with the number of inflorescences as an effect of different electrical conductivity was used, and the paired group (UPGMA) algorithm was applied by using the Euclidean distance to space the cluster. As shown in Figure 5, the hierarchical clustering (*r*° = 0.85) organized the *Viola* × *wittrockiana* seedlings in two main clusters based on the plant growth parameters under the influence of different EC nutrient solutions, separating the Yellow seedlings under 5 mS cm^−1^ EC treatments mainly due to plant’s rapid development, volume and root area. The following major branch of the dendrogram includes the White and Blue seedlings under 5 and 6.5 mS cm^−1^ EC treatments which were clearly differentiated from the others due to their similar growth, shoot and leaves number. The next branch comprises the Yellow seedlings under Control, 2, 3.5, and 6.5 mS cm^−1^ EC treatments which had similar plant growth, root length and moderate number of inflorescences. The last branch comprises the 2 and 3.5 mS cm^−1^ EC treatments form the White and Blue *Viola* seedlings which exhibited similar growth, plant volume and number of inflorescences. As it can be foreseen in the dendrogram, similar results were noticed in the case of White and Blue *Viola* seedlings, which had demonstrated increased plant development compared with control and the Yellow seedlings under the respective treatments (i.e., 5 and 6.5 mS cm^−1^ EC).

The relationship between the main vegetative parts of the seedlings and the inflorescences as affected by the different EC nutrient solutions can be seen in Figure 5. From the present study results, it can be stated that for all three pansies, the growth, number of shoots leaves, inflorescences and root development were significantly influenced by the EC nutrient solutions. Where the seedlings recorded a higher growth, number of shoots and leaves the resulting number of inflorescences was also higher demonstrating the positive effect of electrical conductivity level (i.e., 5 mS cm^−1^ EC) on plant development.

On the basis of the PCA, the first two principal components explained 73% of the data variance, showing a good discrimination of the pansy seedlings.

**Figure 6:**
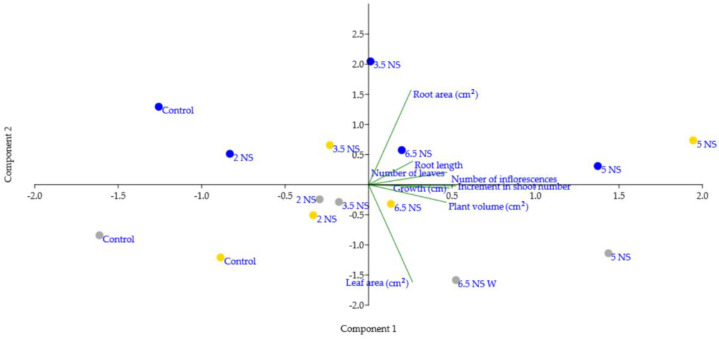
PCA biplot of *Viola* seedlings based on their EC and development. Colored dots represent the type of seedlings (grey—White *Viola* seedlings; blue—Blue *Viola* seedlings; golden—Yellow *Viola* seedlings).

As shown in Figure 6, the analyzed seedlings were clearly differentiated based on electrical conductivity levels and plant growth and development. Thus, in the first upper quadrant the control and 2 mS cm^−1^ EC nutrient solution treatments are displayed with the lowest growth and development in Blue *Viola* seedlings. In the second quadrant, the Blue seedlings with 3.5 mS cm^−1^ EC treatments had lower shoot leaf area and plant volume but higher root length and area. Conversely, the Blue pansies under the 5 and 6.5 mS cm^−1^ EC treatments had similar development and volume, but slightly lower root architecture traits compared with the other treatments. The Yellow *Viola* seedlings under the 5 mS cm^−1^ EC treatments presented similar development with the Blue seedlings, except plant volume and root traits. In the third quadrant, the 5 and 6.5 mS cm^−1^ EC treatments had similar results in terms of plant growth and development in the White and Blue accessions. A higher number of leaves were noticed in White *Viola*, and a higher growth rate and plant development in White *Viola* seedlings under the 5 mS cm^−1^ EC treatments. Conversely, under the 6.5 mS cm^−1^ EC treatments, higher leaf area and root lengths were noticed. In the last quadrant, all tested seedlings had lower shoot number, leaves and inflorescences and slightly higher development with 2 mS cm^−1^ EC treatments similar with those of control.

## 3. Discussion

The results of the present experiment indicate that appropriate EC level of nutrient solutions can have a positive effect on pansy seedlings. Plants require large amounts of macronutrients to carry out cellular functions, K in one amongst these, and stands 3rd on the list of requirement and importance to plants [36]. Macronutrients play a key role in plant development and can be divided into two groups, primary macronutrients (P, K, and N) and secondary macronutrients (Ca, Mg, and S); among these nutrients, N, P, and K are the most crucial elements in plants [22]. K nutrition could optimize water use efficiency and photosynthesis [37]. All the analyzed parameters are important in pansy cultivation; however, the number of inflorescences, the final product, is a key factor in floriculture production.

From the data obtained, it can be concluded that the different electrical conductivity levels did not affect the growth media pH level; only for ‘Delta’ White an increase was recorded at 3.5 mS cm^−1^ EC treatment. In previous studies, it was mentioned that some plants prefer acid conditions, and their growth is increased at a low pH, which results in a greater P uptake [38,39]. As for pansy, the preferred pH level is between 4.8–5.8, which is an acidic soil. High pH can be a major limiting factor even for rice production in saline-–sodic soil [40].

Considering the growth media EC level, significant differences were observed between the treatments and the control. However, the measured EC level of the growth media was lower than the initial electrical conductivity added by the treatments. This could be explained by the fact that the pansy seedlings absorbed a part of the applied nutrient. It is important to mention that in control, half of the applied nutrient was absorbed by the seedlings; on the other hand, 2, 3.5, and 5 mS cm^−1^ EC also absorbed the approximately ~½ part of the solution. However, at 6.5 mS cm^−1^, EC treatments seedlings retained even more of the nutrient solution, almost 75%. High EC levels can be linked to high salinity, which decreases osmotic potential, and can limit the water absorption, therefore limiting seed germination and even inhibit plants’ growth [41]. A previous study demonstrated that a higher level of EC than 2.5 dS m^−1^, caused physiological damage to *Canarium denticulatum* and inhibited the plant development [42]. In a study, it was noticed that, phyto-depurated wastewater with a 4.5 dS m^−1^ improved the pansy fresh weight, water content, plant height and width, and the number of leaves and flowers [43].

Regarding the growth of pansies, significant increases were observed in the treated seedlings; moreover, a significant one was observed in the Yellow seedlings subjected to 5 mS cm^−1^ EC. A previous study reported that biochar with a higher EC level negatively impacted the growth of both pansy and basil [20]. On the contrary, in accordance with our results, some studies suggested that K has a positive effect on the plant growth [44]. Another study showed that greatest growth was recorded at N:K 40:30, which also had a positive effect of the flowering parameters of pansy [45]. Currey et al. [46] demonstrated that cilantro, dill, and parsley can be grown successfully in different ranges of EC levels in hydroponic cultivation.

Increment in shoot number was also positively influenced by the treatments. For all EC levels, shoot number was highly increased compared with control, moreover 5 mS cm^−1^ EC recorded even greater results than the other three treatments. In some cases, the seedlings shoot number was even double compared with control or treated plants.

Considering the leaves, it can be stated that the treatments efficiently increased the leaf number. Thus, in ‘Delta’ Blue and Yellow pansies, a greater increase was recorded at 5 mS cm^−1^ EC, compared with the other treatments. However, 6.5 mS cm^−1^ EC had a higher electrical conductivity level, seedlings leaf number was inhibited, which could be explained that for pansies, a higher level of EC could have a negative effect on leaf appearance. Consistent with the present study results, previously, it was demonstrated that 6.5 ds m^−1^ EC reduced the leaf expansion and individual leaf area in tomato [47]. In a different study, treatment with nutrient solution at lower levels (1 and 2 dS m^−1^) had positive effects in the leaf area and number, whereas under higher nutrient solution (5 and 6 dS m^−1^) treatments a negative effect was observed in all growth characteristics of red perilla [22]. Similarly, treatment with EC 3 dS m^−1^ had a positive effect in the growth and development of red and green perilla, which may be due to the difference in nutrient solution formulas [48].

In the present study, EC treatment had a positive effect in root length and area under the 3.5 mS cm^−1^ EC in Blue pansies and under the 6.5 mS cm^−1^ EC treatments in Yellow seedlings. Previously, it has been demonstrated that increased nutrient solution concentration leads to water absorption inhibition in roots, causing water stress, which results in several physiological changes in plants [49]. In a different study, shoot, root and leaf area ratios increased with increasing nutrient solution application 3.7 dS·m^−1^ [50].

The data obtained shows that electrical conductivity level can have a positive influence on the number of inflorescences, which can be observed from our results. It can be stated that almost all treatments increased the number of flowers, with 5 mS cm^−1^ EC having the best influence on the inflorescences. It is important to mention that 6.5 mS cm^−1^ EC was at a higher level; even so, the inflorescences appeared inhibited compared with 5 mS cm^−1^ EC or similar with control. This could be explained by the fact that pansies act negatively when are irrigated with higher than 5 mS cm^−1^ EC level. In a study, it was demonstrated that 1.6 mS cm^−1^ EC increased the flowering and fruit yield of June-bearing varieties, also suggesting that EC plays an important role in regulating frequency and complexity of inflorescence architecture [51]. Naik et al. [52] determined that 0.8–1 mS cm^−1^ EC yielded the highest quality of flowers of *Cymbidium* hybrids.

## 4. Materials and Methods

### 4.1. Plant Material and Experimental Site

The experiment was conducted between 4 November 2019 and 20 February 2020 in a commercial greenhouse (Blondy) with controlled temperature and humidity conditions, located in Mureș County, Glodeni (46°36′33″ N 24°38′08″ E). The *Viola* × *wittrockiana* ‘Delta’ seeds were obtained from Syngenta (Basel, Switzerland) in three colors, blue, yellow, and white, respectively. The selected *Viola* seedlings are suitable for spring and autumn production and have early, uniform and big flowers. They have a compact/bushy growth and are less liable for elongation.

**Figure 7:**
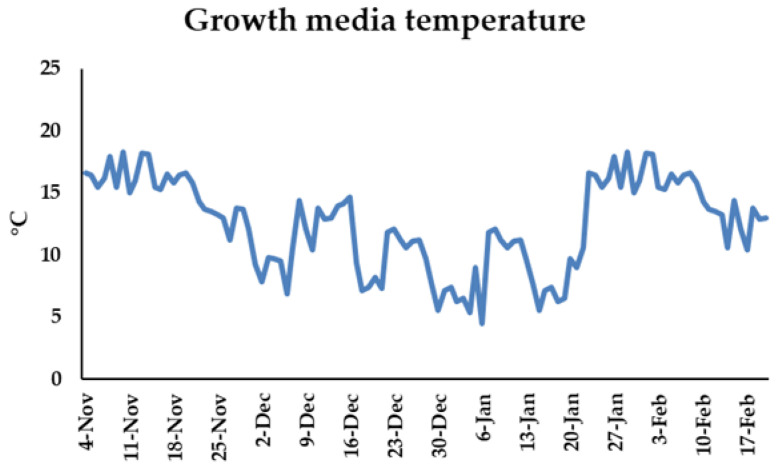
Growth media temperature (°C) during the experiment.

Seeds of the three selected *Viola* seedlings were sown in cell trays (Teku) filled with peat (Danmuld Substrate Latvia), with the fraction between 0–6 mm. After the seedlings were transplanted to pots, 0–20 mm peat was used; and for each peat the pH level varied between 5.5 and 6. The growth media temperature was measured during the experiment with a SM150T Soil Moisture Sensor (Delta-T Devices, Cambridge, UK), and the temperature fluctuated between 4.48 and 18.3 °C (Figure 7).

**Figure 8:**
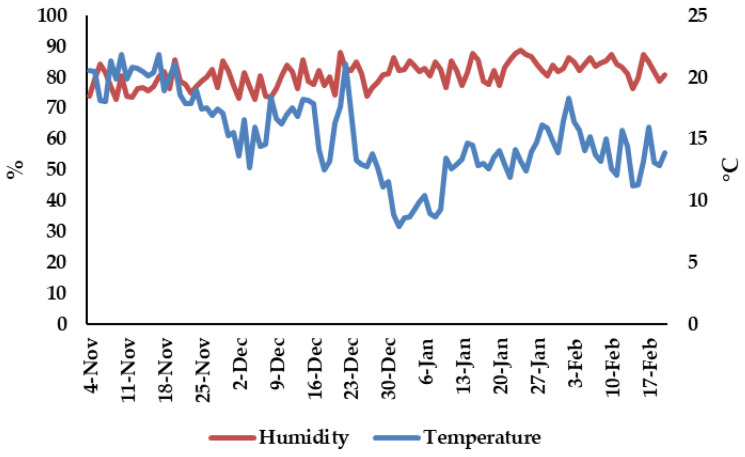
Humidity (%) and temperature (°C) during the experiment.

Humidity and temperature were measured using a Testo 175H1 (Testo Romania, Cluj-Napoca, Romania). Recorded average humidity during the experiment was 80.9%, and the average temperature was 15.19 °C (Figure 8).

**Figure 9:**
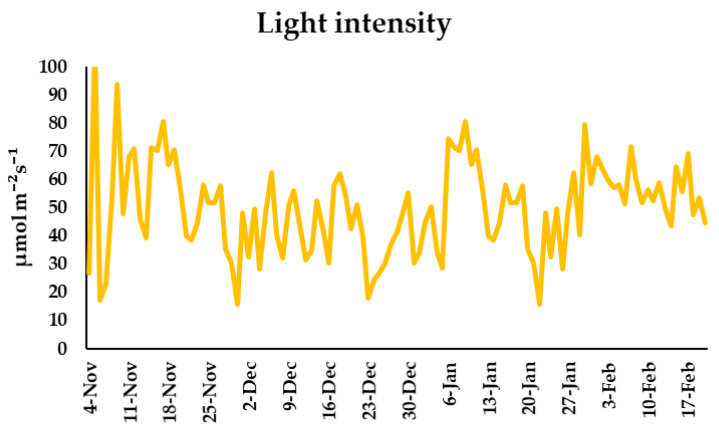
Daily light intensity (µmol m^−^²s^−^¹) during the experiment.

During the greenhouse experiment, the daily light intensity (Xiaomi Flora, China) was measured, the average being 49.82 µmol m^−^²s^−^¹; however, for pansies, it is recommended to ensure more than 34.72 µmol m^−^²s^−^¹ to have an abundant flowering (Figure 9).

### 4.2. Experimental Design

For each *Viola*, 10 seedlings/replicate were selected, with four replicates for each experiment with a total of 200 seedlings. The experiment was designed in a RCBD (Randomized Complete Block Design). The seedlings were irrigated similarly with a nutrient solution with a 1.5 mS cm^−1^ EC, and after they reached three real leaf phenophase, were transplanted individually in 0.3-L pots on 4 November 2019.

Five different levels of electrical conductivity were prepared for the treatments: 2, 3.5, 5, 6.5, and 1 mS cm^−1^ EC, which was considered as the control. To obtain the electrical conductivity (EC) levels stated previously, K predominant nutrient solution was used in the following proportion N:P:K:Mg:Ca 1:0.35:2:0.2:1. Nutrient solution treatments were applied by irrigating the plants twice a week with 1 L per tray solution (1 (control), 2, 3.5, 5, and 6.5 mS cm^−1^ EC).

### 4.3. Data Evaluation/Plant Growth Parameters

The EC (mS cm^−1^) and pH (CyberScan EC/pH–ENVCO) of the growth media was measured when applying the treatments, twice a week. Growth, increment in shoot number, number of leaves, inflorescences, root length, leaves, and root areas were determined just before starting the treatments (time 0 days) and before the end of the treatments (time 109 days), when the plants were fully developed.

### 4.4. Statistical Analysis

The significance of the differences between the treatments was tested by applying one-way ANOVA, at a confidence level of 95%. When the ANOVA null hypothesis was rejected, Tukey’s post hoc test was carried out to establish the statistically significant differences at *p* < 0.05. Cluster analysis and principal component analysis (PCA) were performed using the Paleontological Statistics (PAST) software (Oslo, Norway). Cluster analysis was performed on a Euclidean distance using complete linkage [53].

## 5. Conclusions

The present experiment provides data on the comparison of different levels of EC nutrient solution and their effect on *Viola* × *wittrockiana* growth and development. According to the obtained results, it can be concluded that all treatments (2, 3.5, 5, and 6.5 mS cm^−1^ EC) positively influenced the seedlings by increasing all growth parameters; even the inflorescences appearance was increased when the different electrical conductivity levels were applied. However, the most suitable EC level was the 5 mS cm^−1^ EC, in which case the growth, and number of shoots and leaves was significantly increased, resulting in a compact bushy plant. Furthermore, the highest number of inflorescences was recorded at the same EC level, which is the most important fact in pansy cultivation.

## Figures and Tables

**Table 1:** Effect of nutrient solution electrical conductivity levels on the development of selected *Viola* × *wittrockiana* seedlings.

Sample	Seedlings Growth (cm)	Leaf Area (cm^2^)	Increment in Shoot Number	Number of Leaves	Plant Volume (cm^2^)
**White**					
Control	6.92 ± 1.2 a	18.176 ± 2.5 c	1.41 ± 0.3 a	8.91 ± 2.1 a	115.164 ± 23.4 a
2 EC	8.71 ± 0.8 bc	20.541 ± 3.1 cd	2.22 ± 0.5 b	15.11 ± 2.5 b	145.14 ± 15.8 c
3.5 EC	9.07 ± 0.9 c	19.339 ± 2.3 c	2.56 ± 0.2 b	18.67 ± 3.1 cd	105.473 ± 16.7 a
5 EC	9.65 ± 1.1 d	28.436 ± 3.3 f	4.52 ± 0.9 c	19.5 ± 4.1 d	250.007 ± 22.5 g
6.5 EC	8.89 ± 1.2 c	31.115 ± 1.8 g	2.78 ± 0.3 b	18.4 ± 3.8 cd	231.757 ± 23.4 f
**Blue**					
Control	6.99 ± 1.8 a	14.729 ± 1.2 b	1.23 ± 0.2 a	8.7 ± 2.7 a	128.16 ± 13.3 b
2 EC	8.59 ± 1.7 b	11.294 ± 0.9 a	2.33 ± 0.4 b	14.1 ± 3.3 b	99.38 ± 16.2 a
3.5 EC	8.95 ± 1.9 c	11.084 ± 2.1 a	2.34 ± 0.5 b	17.6 ± 2.9 c	148.602 ± 12.1 c
5 EC	9.83 ± 1.2 d	20.974 ± 1.0 cd	4.61 ± 1.0 c	19.7 ± 3.4 d	201.441 ± 14.9 e
6.5 EC	8.58 ± 1.1 b	22.627 ± 2.6 d	2.39 ± 0.4 b	17.9 ± 2.9 c	179.231 ± 15.7 d
**Yellow**					
Control	8.64 ± 0.7 b	26.966 ± 2.7 f	1.11 ± 0.1 a	8.8 ± 1.5 a	168.847 ± 16.6 d
2 EC	8.61 ± 0.5 b	20.74 ± 2.2 c	2.15 ± 0.1 b	14.6 ± 2.3 b	176.371 ± 12.9 d
3.5 EC	8.97 ± 0.4 c	13.357± 0.6 b	2.35 ± 0.7 b	17.7 ± 4.0 c	185.978 ± 20.5 d
5 EC	9.84 ± 0.6 d	24.036 ± 3.0 be	4.42 ± 0.8 c	19.6 ± 2.8 d	334.38 ± 25.5 h
6.5 EC	8.64 ± 0.5 b	23.209 ± 2.1 de	2.76 ± 0.3 b	17.4 ± 3.1 c	212.128 ± 17.8 f

Different letters indicate significant differences between treatments, according to Tukey’s post hoc test (*p* < 0.05).

## Data Availability

Not applicable!
